# Detection of Pathogens and Ticks on Sedentary and Migratory Birds in Two Corsican Wetlands (France, Mediterranean Area)

**DOI:** 10.3390/microorganisms11040869

**Published:** 2023-03-28

**Authors:** Baptiste Defaye, Sara Moutailler, Benjamin Vollot, Clémence Galon, Gaëlle Gonzalez, Rayane Amaral Moraes, Antoine-Simon Leoncini, Amalia Rataud, Gilles Le Guillou, Vanina Pasqualini, Yann Quilichini

**Affiliations:** 1 UMR CNRS SPE 6134, Université de Corse Pascal Paoli, F-20250 Corte, France; 2 ANSES, INRAE, Ecole Nationale Vétérinaire d’Alfort, UMR BIPAR, Laboratoire de Santé Animale, F-94700 Maisons-Alfort, France; 3 BV’Nat, Expert Indépendant, F-30670 Aigues-Vives, France; 4 ANSES, INRAE, Ecole Nationale Vétérinaire d’Alfort, UMR VIROLOGIE, Laboratoire de Santé Animale, F-94700 Maisons-Alfort, France; 5 Ornithologue de la Réserve Naturelle de l’Étang de Biguglia, Collectivité de Corse, F-20290 Borgo, France; 6 Université Paris Est, ANSES, Laboratory for Animal Health, Epidemiology Unit, F-94700 Maisons-Alfort, France; 7 PUPIPO Project, Laboratoire Eco-Entomologie, F-45000 Orléans, France; 8 Association CHENE, F-76190 Allouville-Bellefosse, France

**Keywords:** arthropod-borne pathogens, Ixodes ricinus, louse flies, migratory birds

## Abstract

Birds are one of the most species-diverse vertebrate groups and are susceptible to numerous hematophagous ectoparasites. Migratory birds likely contribute to the circulation of these ectoparasites and their associated pathogens. One of the many migration paths crosses the Mediterranean islands including Corsica and its wetlands, which are migration stopovers. In our study, we collected blood samples and hematophagous ectoparasites in migratory and sedentary bird populations in two coastal lagoons: Biguglia and Gradugine. A total of 1377 birds were captured from which 762 blood samples, 37 louse flies, and 44 ticks were collected. All the louse flies were identified as *Ornithomya biloba* and all the ticks were from the *Ixodes* genus: *Ixodes* sp. (8.5%), *I. accuminatus/ventalloi* (2.9%), *I. arboricola/lividus* (14.3%), *I. frontalis* (5.7%) and *I. ricinus* (68.6%). Five pathogens were detected: *Anaplasma phagocytophilum, Erhlichia chaffeensis,* and *Rickettsia helvetica* in ticks, and *Trypanosoma* sp. in louse flies. *Ehrlichia chaffeensis* and the West Nile virus were both detected in bird blood samples in Corsica. This is the first report of these tick, louse fly and pathogen species isolated on the bird population in Corsica. Our finding highlights the importance of bird populations in the presence of arthropod-borne pathogens in Corsican wetlands.

## 1. Introduction

Birds are one of the largest vertebrate groups, with approximately 10,000 species, of which approximately 5000 belong to the order Passeriformes [1]. Passerines can be sedentary—a lifestyle more susceptible to being infected by ectoparasites—or migratory—making them potential dispersal agents of ectoparasites and their pathogens along the migration routes [2,3]. The Mediterranean area spans the main migration areas for autumn migration (postnuptial) from Northern Europe to North or sub-Saharan Africa and spring migration (prenuptial) from Africa to Europe. The migration route is mainly defined by two migration paths: one across France, Spain, and Gibraltar, and one across Turkey and Israel [4,5]. However, some migration also covers the Mediterranean islands [4,6,7] such as Corsica.

Migratory birds are frequently present in wetlands, which serve as water spots during migration stopovers, where they may interact with sedentary birds [8]. Wetlands are areas of high levels of interaction between animals and represent one of the main watering and resting areas for them [9,10]. These habitats are important for public health given their role in vector proliferation and pathogen transmission [8,11,12]. The diversity and the prevalence of ectoparasite communities and pathogens in these ecosystems can lead to different health-related threats [13].

Via their migrations routes, birds can play a role in the circulation of numerous pathogens, becoming infected, reservoir, or acting as transport infected vectors. Avian influenza is the perfect example of a large-scale virus infection affecting many birds, including domestic or poultry species. Avian influenza is responsible for numerous outbreaks around the world every year [14,15]. Birds can also transmit bacteria responsible for public or animal health and economic issues such as *Chlamydia psittaci*, the causative agent of psittacosis [14], or numerous tick-borne pathogens [16], vector-borne viruses, e.g., West Nile virus (WNV) and Usutu virus (USUV), or parasites such as *Trypanosoma* spp. and *Hepatozoon* spp. [17]. West Nile virus and USUV are emerging zoonotic pathogens transmitted by mosquitoes that can infect birds, humans, and a wide range of other animal species [14,18,19]. As transport vectors, migratory birds can be involved in the circulation of vectors, such as ticks, which can carry pathogens, e.g., *Borrelia burgdorferi sensus lato*, the causative agent of Lyme borreliosis [20].

Among the arthropods responsible for the circulation of pathogens, ticks, and louse flies can be carried by birds over long distances. Ticks (Ixodida) are the leading vector-borne pathogens in animals and second in humans, just after mosquitoes [21]. They are involved in pathogen transmission and circulation in various types of animals all over the world: by feeding on their host’s blood, ticks harbor and transmit tick-borne pathogens (TBPs) in a natural cycle involving their intermediate and final animal hosts and accidental human hosts [21]. As such, ticks are involved in the transmission of a wide range of bacteria, viruses, and parasites. TBPs are known to be both pathogens of veterinary importance and zoonotic pathogens harmful to humans [22]. Through the dissemination of different tick species, migratory birds can facilitate the spread of a wide range of TBPs [23]. In two tick surveys, 10 species have been recorded in Corsica (Mediterranean island): *Amblyomma variegatum* (sporadically), *Ixodes ricinus*, *Rhipicephalus bursa*, *Rh. sanguineus* s.l., *Rh. annulatus*, *Hyalomma marginatum*, *H. scupense*, *Haemaphysalis sulcata*, *Ha. punctata* and *Dermacentor marginatus* [24,25], but no research has been conducted on tick infestations of birds in Corsica.

Louse flies (superfamily: Hippoboscoidae) are biting flies that infest a large diversity of mammals and birds, and occasionally humans. They are divided into two families: the family Hippoboscoidae targeting birds and mammals and the family Nycteribiidae targeting bats [26]. Even though they have a lower health impact on humans than ticks and mosquitoes, they can have an impact on animals through the blood lost during feeding and the transmission of pathogens such as *Rickettsia* spp., *Bartonella* spp., and *Trypanosoma* spp. in wild ungulates [26,27,28,29]. In birds, louse flies are responsible for the transmission of parasites such as *Haemoproteus* spp. involved in bird mortality and morbidity [30]. Until this study, the only louse flies identified in Corsica were collected on bats and belonged to the family Nycteribiidae.

The aims of this study were (i) to identify and determine the ectoparasite species in birds sampled from Corsican wetlands and (ii) to investigate the potential occurrence of zoonotic and non-zoonotic pathogens in birds and in their ectoparasites in Corsican wetlands. To do so, we restricted our screening to the detection of 34 species and 11 genera of bacteria, viruses, and parasites suspected to be present in Corsica that have an important impact on animal and human health and belong to the main TBPs.

## 2. Materials and Methods

**Sites and animal sampling**

Corsica is the largest French island in the Mediterranean Sea at 15 km from an Italian coast (Sardinia Island) and 160 km to a French coast. The island is characterized by mild Mediterranean climate and a wide spectrum of landscapes, including wetlands (mainly along the coastline), forests, and mountains (up to 2706 m). Corsica is divided into two administrative units (called *départements*: *Haute-Corse* and *Corse du Sud*), with a population of 340,000 inhabitants, which increases nearly 10-fold during the tourist season (summer), especially in the coastal wetlands. These wetlands can be classified into five categories: lagoons, rivers and river mouths, artificial lakes, altitude lakes, and temporary pools. Eastern Corsica is characterized by a particularly high number of coastal wetlands. In this study, all samples were collected in coastal wetlands.

**Figure 1:**
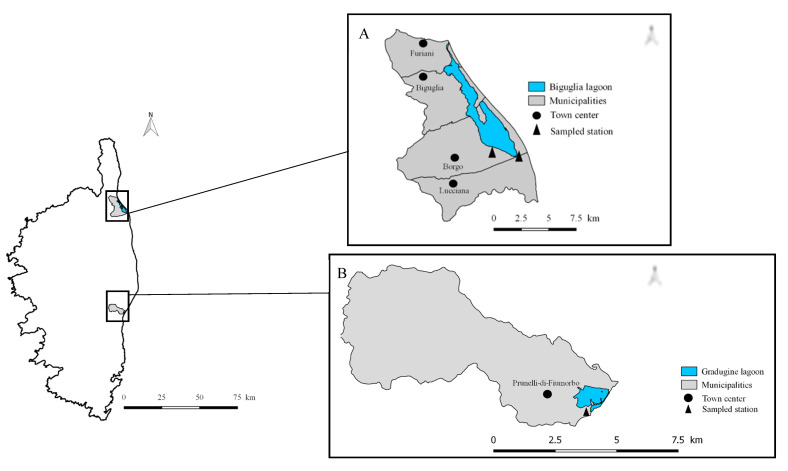
Map of Corsica with the sampled wetland locations (**A**: Biguglia lagoon; **B**: Gradugine lagoon).

A total of 1377 birds were captured during regular banding programs using Japanese nets (2.5 m × 12 m) in two main migration hotspot wetlands on the eastern coast of Corsica, namely the Biguglia and Gradugine lagoons, during autumn migrations in 2019 and 2020; and during spring migration in 2021 (Figure 1). From these birds, 762 blood samples, 37 louse flies, and 35 ticks were collected. All blood samples and ectoparasites were collected by a certified bird bander from the CRBPO (*Centre de Recherches sur la Biologie des Populations d’Oiseaux du Muséum National d’Histoire Naturelle, Paris, France*) in order to minimize as much as possible the animal’s pain, trapping, and handling procedures were approved by the appropriate authorities (authorization no. PP 1062). All blood samples were stored at −80 °C and the ectoparasites were stored in 70% ethanol at −20 °C within a few hours after sampling.

**DNA extraction and PCR pre-amplification**

The ectoparasites were washed in ethanol and distilled water prior to being then morphologically determined to the species level using species identification keys [31,32] for ticks or sent for determination to the entomologist in charge of the PUPIPO project for louse flies. Prior to DNA extraction, ectoparasites were crushed by using microtubes filled with six metal beads using a Fisherbrand Bead Mill 24 homogenizer (Thermo Fisher Scientific, Waltham, MA, USA) at 5500 rpm for 20 s. DNA from ticks was extracted using a Nucleospin Tissue kit (Macherey-Nagel, Düren, Germany). The DNA and RNA in the blood samples were extracted using a FastPrep Ribolyzer (Thermo Fisher Scientific, USA) in BSL3 facilities. All ticks were pooled by species, stage, and sex collected from the same bird (minimum number of ticks = 1, maximum number of ticks = 11). The louse flies were crushed and analyzed individually.

In order to improve the detection of DNA of pathogens, total DNA was preamplified by using the PreAmp Master Mix (Fluidigm, South San Francisco, CA, USA). Primers targeting all pathogens were pooled combining equal volumes of primers (200 nM final each). The experiment was performed with 1 µL PreAmp Master Mix, 1.25 uL pooled primers mix, 1.5 µL distilled water, and 1.25 µL DNA for a 5 µL of final volume. The preamplification cycling was as follows: one cycle at 95 °C for 2 min, 14 cycles at 95 °C for 15 s, and 60 °C for 4 min. At the end, the samples were diluted 1:10 as performed by [33]. Preamplified DNA and RNA samples were stored at −20 °C until further use.

**Assay design**

Pathogens and their targeted genes were *Anaplasma* spp. (16S), *A. marginale* (Msp1b), *A. phagocytophilum* (Msp2), *Borrelia* spp. (23S), *Bo. Burgdorferi* s.s. (rpoB), *Bo. Afzelii* (flagellin), *Bo. Miyamotoi* (glpQ), *Bo. Lusitaniae* (rpoB), *Bo. Spielmanii* (flagellin), *Chlamydiaceae* (23S) [34], *Rickettsia* spp. (gltA), *R. slovaca* (23S-5S ITS), *R. helvetica* (23S-5S ITS), *R. aeschlimanii* (23S-5S ITS), *R. massiliae* (23S-5S ITS), *R. conorii* (23S-5S ITS), *C. burnetii* (idc, IS1111), *N. mikurensis* (ARN16S), *Bartonella* spp. (ssrA), *Ba. Henselae* (pap31), *Ba. Quintana* (bqtR), *Ehrlichia* spp. (16S), *E. ruminantium* (dsb), *Hepatozoon* spp. (18S), *To. Gondii*, *B. microti* (CCTeta), *B. bigemina* (ARN 18S), *B. vogeli* (hsp70), *B. caballi* (rap1), *B. bovis* (CCTeta), *B. ovis* (ARN18S), *B. divergens* (hsp70), *T. equi* (Ema1), *T. annulata* (ARN 18S), *Leishmania* spp. (hsp70), *L. infantum* (ITS), and African swine fever virus (Vp72). The primers and probes selected for these pathogens were found in the literature [33,35,36] except for Chlamydiaceae. The primer/probe sets created in this study are listed in Table 1.

**DNA amplification, microfluidic real-time PCR and RT-PCR**

The detection of the targets of vector-borne pathogens was performed on preamplified DNA using the BioMark^TM^ real-time PCR system for high-throughput microfluidic real-time PCR amplification (Fluidigm, CA, USA). Fluidigm chips can handle 2304 real-time PCR reactions, i.e., from 48 PCR mixes for 48 samples placed into individual wells prior to transfer into individual chambers for the reaction. The thermal cycling conditions were 50 °C for 2 min, 95 °C for 10 min, and 40 cycles at 95 °C for 15 s and 60 °C for 1 min. One negative water control, one inhibitory molecule control (*E. coli* EDL933 strain), and one DNA extraction control (bird-specific primers) were added to each chip. Specific primers and probes of *E. coli* were used. All RNA extracts underwent real-time RT-PCR following the protocol described previously [37] for the detection of WNV and USUV.

**Confirmation of detected pathogens**

Pathogen detection was confirmed using nested PCR or real-time PCR targeting a gene other than that used for the microfluidic experiment. Primer/probe sets were selected from the literature when available or newly designed in this study. The positive sequences after gel migration from the nested PCR product were sent for sequencing at Eurofins MWG Operon (Cologne, Germany) and assembled using BioEdit software (Ibis Biosciences, Carlsbad). An online BLAST (National Center for Biotechnology Information) was used to identify the sequenced organisms. All the target genes and primer sequences from the literature are described in [33,34,35]. The primer/probe sets created for this publication are listed in Table 2.

**Birds Behavior**

In our research, the birds captured and sampled had different behavior, which had already been defined in previous studies [38]. For purpose of better legibility of the birds’ behavior, we decided in our study to regroup them into three behavior categories: sedentary, migratory, and sedentary/migratory. They, respectively, regroup former breeders, introduced breed, and resident breeders for sedentary birds; accidental visitors, breeding visitors, occasional breeders, passage migrants, and winter visitors for migratory birds; birds that have behaviors from these two groups will be defined as sedentary/migratory birds. However, for the sake of accuracy, the terms defined by Thibault and Bonaccorsi will be kept in Supplementary Table S1.

## 3. Results

### 3.1. Ectoparasite Carriers of Pathogens in Birds

From the ectoparasites sampled in wild birds, 37 louse flies were collected on 35 birds representing 2.5% of the birds captured with an infestation intensity of 1.06 (louse flies per infested birds). All were collected on the migratory bird species *Hirundo rustica* at a rate of about 10.6% of the captured individuals. All louse flies collected were adult *Ornithomya biloba* (Table 3 and Table 4) and 94.3% of them were collected near the Gradugine Lagoon and 5.7% in the Biguglia Lagoon in the autumn season. Of these, 67.6% were female and 32.4% were male; only one was positive for *Trypanosoma* spp., for which the sequencing was unfortunately not successful. All other louse flies were negative for all the other targeted pathogens (Table 3 and Table 4).

The other ectoparasites sampled on wild birds were 44 ticks, sampled from 24 birds (1.7% of the birds captured). They were collected on bird species with various behavior during the spring session of 2021 (Table 3): 36.3% from *Erithacus rubecula* (sedentary/migratory), 22.7% from *Saxicola rubicola* (sedentary/migratory), 18.2% from *Cettia cetti* (sedentary), 6.9% from *Turdus merula* (sedentary/migratory), 4.5% from *Acrocephalus arundinaceus* (migratory), 4.5% from *Prunella modularis* (migratory), 2.3% for *Luscinia megarhyncos* (migratory), 2.3% from *Parus major* (sedentary) and 2.3% from *Sylvia atricapilla* (sedentary/migratory) (Table 3 and Table 4). All collected ticks were from the *Ixodes* genus: *Ixodes* spp. (8.5%), *I. accuminatus/ventalloi* (2.9%), *I. arboricola/lividus* (14.3%), *I. frontalis* (5.7%), and *I. ricinus* (68.6%), with 97.1% of the ticks sampled in the Biguglia Lagoon wetlands and 2.9% in the Gradugine Lagoon. A total of 27 different pools were constituted. Among the pools, one pool of nymphs collected on *S. atricapilla* in the Biguglia Lagoon was positive for *A. phagocytophilum.* Another pool of larvae collected on *E. rubecula* from the Biguglia Lagoon was positive for *Ehrlichia* spp. which appears to be closely related to an *E. chaffeensis* sample from goat’s blood in China (GenBank: KX505292.1). Three other pools (one of larvae, one of nymphs, and one of adults) were positive for *R. helvetica* collected from one *C. cetti* and two *E. rubecula* in the Biguglia Lagoon. One pool of larvae from *L. megarhyncos*, one pool of 10 engorged nymphs from *Sax. Rubicola,* and another pool of engorged nymphs from *T. merula* were positive for *Rickettsia* spp. (Table 3). Unfortunately, the sequencing of *A. phagocytophilum*, *R. Helvetica,* and *Rickettsia* spp. was not successful.

### 3.2. Pathogens Detected in Blood Samples

From bird blood samples, two pathogens were detected. West Nile virus was found in two adult *Parus major* (sedentary/migratory) from the Gradugine Lagoon during autumn migration in 2019 (Table 3). Unfortunately, sequencing was unsuccessful. Regarding TBPs, one *Erithacus rubecula* (sedentary/migratory) from the Biguglia Lagoon sampled in spring 2021 was positive for *Ehrlichia chaffeensis* which appears to be closely related to a sample from goat’s blood in China (GenBank: KX505292.1). The same passerine individual harbored tick larvae positive for *E. chaffeensis* (see above, Table 4).

## 4. Discussion

### 4.1. Bird Ectoparasites and Their Pathogens

#### 4.1.1. Louse Flies

The Hippoboscidae family belongs to the superfamily Hippoboscoidea, which groups numerous bloodsucking fly families. This family has a worldwide distribution and can be found in both mammals and birds [39]. In Europe, different genera have been detected, such as *Liptotena* on cervids, *Melophagus* on domestic ruminants, and *Ornithomya* on birds, mainly on passerines [40]. Two *Ornithomya* species (*O. avicularia* and *O. chloropus*) have a wide distribution and, for example, have been reported in bird nests in Norway [41]. Both species have also been found in the Czech Republic along with a third species *O. biloba*, on birds [42]. Although this genus is mainly a nest parasite, it is also observed during summer and autumn migration [41,42]. In our study, we only found one species: *O. biloba* collected on *Hirundo rustica* in autumn 2020. This species is mainly present on Hirundinidae passerines, such as *H. rustica* [40,43]. This species has already been reported in France during a survey of Hippoboscidae carried out by the PUPIPO project [44] on different hosts; it was the second-most frequent louse fly species and was found nearly exclusively on *H. rustica*. However, the survey of the PUPIPO project miss the information on the situation on the bird population in Corsica, only louse flies from *Nycteribiidae* bats were collected in the PUPIPO project. This study is the first report of bird louse flies in Corsica.

The Hippoboscidae family may be responsible for the circulation of various pathogens; however, to date, there is little information on them [45]. Nevertheless, one study has suggested that cervid louse flies *Liptotena cervi* may be involved in the mechanical transmission of *A. phagocytophilum* [46]. Similarly, in birds, the genus *Ornithomya* is involved in the transmission of *Haemoproteus* and *Trypanosoma* parasites to their avian host [47,48]. In our study, we most probably detected *Trypanosoma* spp. DNA in one of the louse flies collected, which is the first record of carriage for Corsica. Although the species could not be identified, as it was in a study in the Czech Republic where a wide range of *Trypanosoma* spp. was reported in *Ornithomya* louse flies [49]. Our study demonstrates the occurrence of infected louse flies in Corsica—which are either vectored by migratory birds or migratory birds are infested by infected Corsican louse flies. However, the absence of louse flies on sedentary birds cannot exclude the possibility that louse flies are transmitted between migratory birds and sedentary birds. Further research is needed on louse flies to better determine their role in the circulation of pathogens and their impact on bird and human health in Corsica.

#### 4.1.2. Ticks

Hard ticks (Ixodidae) are vectors of several pathogens, such as *Rickettsia* spp., *Borrelia* spp., and Crimean-Congo Hemorrhagic Fever virus (CCHFV) [50,51,52]. In Europe and the Mediterranean, most ticks found on avian species are immature stages from the genus *Hyalomma*, *Ixodes* ticks of all stages and, sporadically, immature stages from the genera *Haemaphysalis* and *Rhipicephalus* [52,53]. However, to date, no information is available on the ticks infesting avian species in Corsica. In our study, we found ticks exclusively from the *Ixodes* genus: *Ixodes* spp., *I. accuminatus/ventalloi*, *I. arboricola/lividus*, *I. frontalis,* and *I. ricinus*. These ticks are frequently found on passerine species among other tick genera, such as *Hyalomma* [2,53,54]. All ticks in our study were sampled on sedentary birds. The *Erithacus rubecula* represented the highest proportion of infested birds (36.3%), on which a total of three different *Ixodes* species were found: *I. arboricola*/*lividus, I. frontalis* and *I. ricinus.* This latter species was the most frequently detected tick species, accounting for 68.8% of the ticks population on *E. rubecula* and 54.5% of the total of ticks collected on other wild birds. Whether migratory or sedentary (as shown in the Corsican wetlands in this study), *E. rubecula* appears to be an important host for immature tick stages, particularly for *Ixodes* ticks. In various countries, *E. rubecula* is the main *Ixodes* tick host; for example, Denmark shows an even higher infestation intensity (between 1.93 and 3 ticks per bird) than in our study (1.45 ticks per bird) [55]. Similarly, other countries show heavy bird tick infestations, such as Latvia, with a mean *Ixodes* tick infestation intensity of 2.4 ticks per bird, or Italy, with a prevalence of 4.5% on *E. rubecula* [2,53]. However, in these cited countries, only migratory *E. rubecula* individual tick-infested were considered migratory, in contrast to Corsica, where *E. rubecula* was considered both sedentary and migratory birds [55]. This bird species is also the main host of the immature stages of *I. frontalis* ticks in Turkey [56]. However, *E. rubecula* is not the only passerine infested by *Ixodes* ticks, which target a wide spectrum of passerines, both sedentary and migratory [52,55,57,58]. On the other hand, the bird species with the highest infestation rate was *Cettia cetti* (2.66 ticks per bird), which is a ground-feeding sedentary bird in Corsica. This high infestation intensity may be linked to the ground-feeding behavior of *C. cetti* which makes it a more susceptible target of ticks [23]. Our results demonstrate the potential importance of the sedentary birds in the stabilization of the *Ixodes* tick population in Corsican wetlands but also suggest possible tick circulation between the sedentary and migratory birds of the same species that co-occur in Corsican wetlands [55,59]. Determining the mechanism of tick circulation requires a comparison of tick infestation during migration and non-migration.

A total of three pathogen genera were found in bird ticks: *Anaplasma* spp., *Ehrlichia* spp., and *Rickettsia* spp. One *Anaplasma* species was found: *A. phagocytophilum* in an *I. ricinus* nymph pool collected from *Sylvia atricapilla*. *A. phagocytophilum* is a worldwide intracellular bacterium mainly transmitted by *Ixodes* ticks, leading to tick-borne fever in a wide range of hosts and to human granulocytic anaplasmosis in humans [60]. In birds, this pathogen has already been reported in *I. frontalis* and *I. ricinus* ticks sampled from four bird species captured in our study: *E. rubecula*, *Luscinia megarhynchos*, *S. atricapilla* and *Turdus merula* [61]. It has also been detected in the spleen of *T. merula* from the Netherlands; furthermore, the presence of *A. phagocytophilum* in North America is often documented in birds and their ticks [59,62,63]. More recently, a different variant was reported from a Greek island in an *Ixodes* tick infesting a *Lanius senator senator* [64]. In Corsica, *A. phagocytophilum* has been mainly reported in *I. ricinus*, but it has also been detected in the tick species *Dermacentor marginatus*, *Haemaphysalis. punctata, Hyalomma marginatum, Hy scupense, Rhipicephalus bursa* and *Rh. sanguineus* s.l. collected on domestic and wild mammals [36,65]. To our knowledge, this is the second detection of *A. phagocytophilum* in ticks collected from sedentary birds in Corsica [36]. However, due to the low prevalence of *A. phagocytophilum* plus the possibility of trans ovarian transmission in ticks, it is more likely a sporadic detection in sedentary birds in Corsica [66]. Nevertheless, our study clearly indicates the potential presence of this pathogen in tick-infested sedentary birds in Corsica, although confirmation of this presence requires isolating the pathogen directly from birds.

The *Ehrlichia* genus is closely related to *Anaplasma*; we detected one species: *E. chaffeensis*. It was detected from a pool of *I. ricinus* larvae collected on *E. rubecula*. *Ehrlichia chaffeensis* is a tick-borne intracellular bacterium causing human monocytotropic ehrlichiosis, but is usually found in *Amblyomma americanum* ticks and is mainly present in the United States [67,68]. Although the *Ehrlichia* genus has already been observed in bird ticks [69,70], this is the first detection of *E. chaffeensis* in bird ticks in the Mediterranean Rim and in Corsica where it has not previously been reported in any animal population. The predominance of *Ixodes* ticks as hosts of *E. chaffeensis* in this study supports the previous suggestion that birds are potential reservoir hosts for this bacterial species. However, the isolation of the pathogens and serological analysis of bird sera are required for confirmation. Further analyses on *E. chaffeensis* are still needed in the bird tick population to determine the actual status of this pathogen in the Corsican wetlands.

Six tick pools were found positive for *Rickettsia* spp. Of these six pools, three were positive for *R. helvetica*. Each of the three pools was from different *Ixodes* species. *Rickettsia helvetica* is a tick-borne rickettsia belonging to the spotted fever group (SFG) associated with symptoms such as fever, myalgia, and meningitis. It has a nearly worldwide distribution and is mainly transmitted by *Ixodes* ticks [71,72]. In birds, it has been detected in 70% of the immature ticks from *I. ricinus* collected on passerines in Poland, 21.6% of *I. frontalis* and *I. ricinus* ticks from birds in the Netherlands, and in 5.6% of *I. ricinus* ticks collected on migratory birds in Sweden [73,74,75]. In Hungary, research suggests that birds are a potential reservoir for *R. helvetica*, with the detection of this bacterial species in 51.4% of the *I. ricinus* and *Haemaphysalis* ticks collected on passerines and also in 4.7% of the birds [76]. In the Mediterranean area, it has been found in Spain with a prevalence of 6% of *I. ricinus* ticks [54]. In our research, we detected the presence of *R. helvetica* for the first time with a prevalence of 11.1% in tick-infested birds. However, in Corsica, *R. helvetica* has already been reported in *I. ricinus* ticks collected on cattle [36].

### 4.2. Pathogens Found in Birds’ Blood

Another objective of the research was to screen for pathogens in bird blood samples. We found two pathogens: one virus, WNV, and one bacterium *E. chaffeensis*.

In birds, an Anaplasmacetae closely related to *E. chaffeensis* has been reported in tissue and blood samples from a *Turdus philomelo* in Hungary [77]. It was also found in the liver of a *Phasianus colchicus* in China [78]. In Corsica, *E. chaffeensis* has not previously been detected in animal samples. Furthermore, the *E. rubecula* individual found positive was also infested with infected *I. ricinus* larvae (*E.* chaffeensis-positive larvae). Therefore, *E. chaffeensis* may circulate in sedentary and migratory birds in the Biguglia Lagoon in Corsica. This is the first Corsican record of this bacterium in birds and their ticks. Further analyses are still needed to confirm if this is just a sporadic infection or if the infection of more ticks collected on sedentary birds suggests a more frequent presence of this pathogen in the sedentary bird population in Corsican wetlands. Furthermore, confirmation of the presence of this pathogen requires the isolation of the pathogen itself.

The second pathogen identified here was the West Nile virus found in two *Parus major* individuals in the Gradugine Lagoon. This virus is maintained in a bird–mosquito–bird transmission cycle. It can also infect mammals as humans and equines and induce diseases ranging from mild illness to severe neurological disorder [79]. In the Mediterranean area, equine and human outbreaks are reported every year across the Mediterranean Rim, including in Algeria, France, Greece, Italy, Israel, Spain, and Tunisia [80,81]. On Mediterranean islands, it has already been found in Cyprus with a prevalence of 1.3% in birds, 4.5% in human blood donors [82], a human case in Sardinia [83], 1.7% in chicken blood in Crete [84] and, in Corsica, with a prevalence of 9.4% in horses and 8.4% in dogs [18]. In our research, we found WNV RNA in two sedentary *P. major* adults with a prevalence of 0.3% in the wild bird blood samples. This is the first record of WNV RNA in the Corsican bird population. Our results complement previous records of WNV-positives horses and dogs and appear to confirm the presence of the virus in Corsica [85]. Further analyses of WNV are required to determine its actual impact on the bird and mosquito populations involved in its transmission in Corsica. For both bird pathogens, comparative analysis between the migratory and sedentary birds can help to determine the possible circulation of those pathogens between sedentary and migratory birds.

However, some expected pathogens were not detected in our survey. It is particularly the case for the parasite order Haemosporida composed of the *Haemoproteus* spp., *Leucyozoon* spp., and *Plasmodium* spp. which are genera known to provoke avian malaria which can be found worldwide [86,87,88]. It is even more surprising that this order was highly reported in a survey from an island close to Corsica, Sardinia where more than 50% of the 217 birds analyzed were positive [89]. Thus, the detection of this order was expected however this can be explained by the low efficacity of the design of our primers. A study focused on the Haemosporida order by using the same design as in Sardinia study [89] could allow a better view of the Haemosporida order in Corsica.

In conclusion, our results show the importance of the research on pathogens from birds and their ectoparasites. It is allowing the monitoring of known pathogens such as the West Nile virus in Corsica, and also in the monitoring and discovery of new pathogens in the Corsican islands such as *Erhlichia chaffeensis* and *Rickettsia helvetica*.

## Figures and Tables

**Table 1:** List of pathogens, targets, and primer/probe sets created for microfluidic real-time PCR screening.

Pathogens	Name	Sequence	Length (Nucleotide)	Gene	Gene References
Aujeszky’s disease virus	Aujv_gp50_F	CTTTATCGAGTACGCCGACTG	225	gp50	Y14834.1
	Av_gp50_R	AACGGGCACTCTTGCCCC			
	Av_gp50_P	CAGATCTTTGGGCGCTGCCGGC			
*Haemoproteus* spp.	Hae_cytB_F	ATATGCATGCTACTGGTGCTAC	240	cytochrome B	AF465579.1
	Hae_cytB_R	CAAATCCATGAAACAAGTCCAGG			
	Hae_cytB_P	CGGTTGCACCCCAGAAACTCATTGAC			
*Leptospira* spp.	Lep_Lipl32_F	CTCTATGTTTGGATTCCTGCC	158	LipL32	MK541891.1
	Lep_Lipl32_R	CCAAGTATCAAACCAATGTGGC			
	Lep_Lipl32_P	ATTGATTTTTCTTCTGGGGTAGCCGCTTTG			
*Leucocytozoon* spp.	Leu_cytB_F	GGGTTATGTCTTACCATGGGG	177	cytochrome B	KF717066.1
	Leu_cytB_R	AATTGCTAGTGCTACGAATGGG			
	Leu_cytB_P	AAATGAGTTTTTGGGGAGCAACCGTTATTAC			
*Plasmodium* spp.	Pla_ssrRNA_F	ATATAGAAACTGCGAACGGCTC	339	ssu rRNA	MK650620.1
	Pla_ssrRNA_R	TTTCTCAGGCTCCCTCTCC			
	Pla_ssrRNA_P	CTCTAATTCCCCGTTACCCGTCATAGC			
*Rickettsia monacensis*	Ric_mon_F	CTCGGTGCCGGTACTTTAAAC	192	ompB	KU961543.1
	Ric_mon_R	GAGCACCGCCAATAAGAGC			
	Ric_mon_P	AGTGCCGATGCAAATACTCCGGTGAC			
*Trypanosoma* spp.	Try_18SRNA_F	GTAATTCCAGCTCCAAAAGCG	178	18S rRNA	EU596263.1
	Try_18SRNA_R	TCAGGAAGGAACCACTCCC			
	Try_18SRNA_P	ACCTCAAGGGCATGGGTCACCAATCC			

**Table 2:** List of pathogens, targets, and primer/probe sets used for species confirmation using real-time PCR.

Pathogens	Name	Sequence	Length (Nucleotide)	Genes	Genes References
*Aujeszky’s disease virus*	Aujv_gp50_F2	AACATCCTCACCGACTTCATG	158	gp50	Y14834.1
	Av_gp50_R2	CTGGTAGAACGGCGTCAGG			
	Av_gp50_P2	AATCGCATCACGTCCACGCCCCC			
*Haemoproteus* spp.	Hae_cytB_F2	CCTTGGGGTCAAATGAGTTTC	231	cytochrome B	AF465579.1
	Hae_cytB_R2	AAGCCGTATCATATCCTAAAGG			
	Hae_cytB_P2	CCTGGACTTGTTTCATGGATTTGTGGAGG			
*Leucocytozoon* spp.	Leu_cytB_F2	GAGTTTCTGGGGAGCTACTG	197	cytochrome B	KU842391.1
	Leu_cytB_R2	GGATTAGTGCTACCTTGAATATG			
	Leu_cytB_P2	TGAATAAATACAATTGCTAGTGCTACGAATGG			
*Plasmodium* spp.	Pla_ssrRNA_F2	TCGAGTTTCTGACCTATCAGC	264	ssrRNA	MK650620.1
	Pla_ssrRNA_R2	AGACTTGCCCTCCAATTGTTAC			
	Pla_ssrRNA_P2	TGGCCTTGCATTGTTATTTCTTGTCACTACC			
*Trypanosoma* spp.	Try_18SRNA_F2	CAACACGGGGAACTTTACCAG	141	18SRNA	EU596263.1
	Try_18SRNA_R2	ATCCTACTGGGCAGCTTGG			
	Try_18SRNA_P2	CAGGGTGAGGATTGACAGATTGAGTGTTC			

**Table 3:** Positive samples collected on birds in two Corsican lagoons. ^‡^ Ap: *Anaplasma phagocytophilum*, Ec: *Ehrlichia chaffeensis*, R: *Rickettsia* spp., Rh: *Rickettsia helvetica*, T: *Trypanosoma* spp. and WNV: West Nile virus).

Bird Species	Lagoon	Blood Samples	*Ixodes* spp. Pools/Positive Pools			*I. accumanitus/ventalloi* Pools/Positive Pools			*I. arboricola/lividus* Pools/Positive Pools			*I. frontalis* Pools/Positive Pools			*I. ricinus* Pools/Positive Pools			*O. biloba* Individuals/Positive Individuals
			Adult	Nymph	Larva	Adult	Nymph	Larva	Adult	Nymph	Larva	Adult	Nymph	Larva	Adult	Nymph	Larva	Adult
Sedentary																		
*Parus major*	Biguglia, Gradugine	80/2 ^WNV‡^	-	-	-	-	-	-	-	1/0	-	-	-	-	-	-	-	-
Migratory																		
*Acrocephalus arundinaceus*	Biguglia, Gradugine	18	-	-	-	-	-	-	-	-	1/0	-	-	-	-	1/0	-	-
*Hirondo rustica*	Biguglia, Gradugine	177	-	-	-	-	-	-	-	-	-	-	-	-	-	-	-	37/1 ^T‡^
*Luscinia megarhynchos*	Biguglia, Gradugine	15	-	-	-	-	-	-	-	-	-	-	-	-	-	-	1/1 ^R‡^	-
*Prunella modularis*	Biguglia, Gradugine	1	-	-	-	-	-	-	-	-	-	-	1/0	-	-	1/0	-	-
Sedentary/Migratory																		
*Cettia cetti*	Biguglia, Gradugine	95	-	-	-	1/1 ^Rh‡^	-	-	-	-	-	-	-	-	-	3/0	-	-
*Erithacus rubecula*	Biguglia, Gradugine	68/1 ^Ec^	-	1/0	1/0	-	-	-	-	-	1/0	-	-	1/1 ^Rh‡^	1/0	4/1 ^Rh‡^	3/1 ^Ec‡^	-
*Saxicola rubicola*	Gradugine	1	-	-	-	-	-	-	-	1/1 ^R‡^	-	-	-	-	-	-	-	-
*Sylvia atricapilla*	Biguglia, Gradugine	148	-	-	-	-	-	-	-	-	-	-	-	-	-	1/1 ^Ap‡^	-	-
*Turdus merula*	Biguglia, Gradugine	17	-	-	1/0	-	-	-	-	-	-	-	-	-	-	2/1 ^R‡^	-	-
**Total**		**762/2 ^WNV‡^, 1 ^Ec‡^**	**-**	**1/0**	**2/0**	**1/1 ^Rh‡^**	**-**	**-**	**-**	**2/1 ^R‡^**	**2/0**	**-**	**1/0**	**1/0**	**1/0**	**11/1 ^Ap‡^, 1 ^R‡^, 1 ^Rh‡^**	**4/ 1 ^Ec‡^, 1 ^R‡^**	**37/1 ^T‡^**

**Table 4:** Ectoparasites (ticks and louse flies) infesting passerine birds.

Bird Species	Tick Species															Louse Fly Species	Number of Ectoparasites (Number of Infested Birds)	Mean Infestation Intensity
	*Ixodes* spp.			*Ixodes accuminatus/ventalloi*			*Ixodes arboricola/lividus*			*Ixodes frontalis*			*Ixodes ricinus*			*Orythomya. biloba*		
	Adults	Nymphs	Larvae	Adults	Nymphs	Larvae	Adults	Nymphs	Larvae	Adults	Nymphs	Larvae	Adults	Nymphs	Larvae	Adults		
*Acrocephalus arundinaceus*	-	-	-	-	-	-	-	-	1	-	-	-	-	1	-	-	2 (2)	1.0
*Cettia cetti*	-	-	-	1	-	-	-	-	-	-	-	-	-	7	-	-	8 (3)	2.66
*Erithacus rubecula*	-	1	1	-	-	-	-	-	2	-	-	1	1	7	3	-	16 (11)	1.45
*Hirundo rustica*	-	-	-	-	-	-	-	-	-	-	-	-	-	-	-	37	37 (35)	1.06
*Luscinia megarhyncos*	-	-	-	-	-	-	-	-	-	-	-	-	-	-	1	-	1 (1)	1.0
*Parus major*	-	-	-	-	-	-	-	1	-	-	-	-	-	-	-	-	1 (1)	1.0
*Prunella modularis*	-	-	-	-	-	-	-	-	-	-	1	-	-	1	-	-	2 (2)	1.0
*Saxicola rubicola*	-	-	-	-	-	-	-	10	-	-	-	-	-	-	-	-	10 (1)	10
*Sylvia atricapilla*	-	-	-	-	-	-	-	-	-	-	-	-	-	1	-	-	1 (1)	1
*Turdus merula*	-	-	1	-	-	-	-	-	-	-	-	-	-	2	-	-	3 (2)	1.5

## Data Availability

The authors confirm that the data supporting the findings of this study are available within the article and its Supplementary Material. Raw data that support the findings of this study are available from the corresponding author, upon reasonable request.

## Supplementary Materials

The following information can be downloaded at: https://www.mdpi.com/article/10.3390/microorganisms11040869/s1, Table S1: Samples and positive samples collected on birds in two Corsican lagoons.
