# Supplementary material for: Detection of Pathogens and Ticks on Sedentary and Migratory Birds in Two Corsican Wetlands (France, Mediterranean Area)
Source: Microorganisms. 2023 Mar 28;11(4):869. doi: 10.3390/microorganisms11040869 (PMC10141976; doi:10.3390/microorganisms11040869)
Supplement: Supplementary file 1 [file microorganisms-11-00869-s001.zip › microorganisms-2279694-supplementary.pdf]

Supplementary Table S1: Samples and positive samples collected on birds in two Corsican lagoons. (†Av: Accidental visitor, FB: Formal breeder, IB: introduce breeder, MB: Breeding visitor, OB: Occasional breeder, PM: Passage migrant, RB: Resident breeder, WV: Winter visitor ; ‡Ap: *Anaplasma phagocytophilum*, Ec: *Ehrlichia chaffeensis*, R: *Rickettsia* spp., Rh: *Rickettsia helvetica*, T: *Trypanosoma* spp. and WNV: West Nile virus)

Bird species	Behavior †	Lagoon	Blood samples	*Ixodes* spp. pools/positive pools			*I. accumanitus/ventalloi* pools/positive pools			*I. arboricola/lividus* pools/positive pools			*I. frontalis* pools/positive pools			*I. ricinus* pools/positive pools			*O. biloba* individuals/positive individuals
				Adults	Nymphs	Larvae	Adults	Nymphs	Larvae	Adults	Nymphs	Larvae	Adults	Nymphs	Larvae	Adults	Nymphs	Larvae	Adults
Sedentary																			
Accipiter nisus	RB	Gradugine	1	-	-	-	-	-	-	-	-		-	-	-	-		-	-
Aegithalos caudatus	RB	Biguglia, Gradugine	0	-	-	-	-	-	-	-	-	-	-	-	-	-	-	-	-
Chloris chloris	RB	Gradugine	2	-	-	-	-	-	-	-	-	-	-	-	-	-	-	-	-
Curruca melanocephala	RB	Biguglia, Gradugine	15	-	-	-	-	-	-	-	-	-	-	-	-	-	-	-	-
Cyanistes caeruleus	RB	Gradugine	1	-	-	-	-	-	-	-	-	-	-	-	-	-	-	-	-
Garrulus glandarius	RB	Cradugine	1	-	-	-	-	-	-	-	-	-	-	-	-	-	-	-	-
Parus major	RB	Biguglia, Gradugine	80/2$^{WNV ‡}$	-	-	-	-	-	-	-	1/0	-	-	-	-	-	-	-	-
Sturnus unicolor	RB	Biguglia	1	-	-	-	-	-	-	-	-	-	-	-	-	-	-	-	-
Migratory																			
Acrocephalus arundinaceus	MB PM	Biguglia, Gradugine	18	-	-	-	-	-	-	-	-	1/0	-	-	-	-	1/0	-	-
Acrocephalus schoenobaenus	PM	Biguglia, Gradugine	9	-	-	-	-	-	-	-	-	-	-	-	-	-	-	-	-

Acrocephalus scirpaceus	MB PM	Biguglia, Gradugine	33	-	-	-	-	-	-	-	-	-	-	-	-	-	-	-	-
Anthus spinoletta	MB PM WV	Gradugine	1	-	-	-	-	-	-	-	-	-	-	-	-	-	-	-	-
Anthus trivialis	PM	Gradugine	5	-	-	-	-	-	-	-	-	-	-	-	-	-	-	-	-
Curruca cantillans	MB PM	Biguglia, Gradugine	6	-	-	-	-	-	-	-	-	-	-	-	-	-	-	-	-
Curruca communis	PM OB	Biguglia, Gradugine	1	-	-	-	-	-	-	-	-	-	-	-	-	-	-	-	-
Emberiza schoeniclus	PM WV	Biguglia, Gradugine	3	-	-	-	-	-	-	-	-	-	-	-	-	-	-	-	-
Hippolais ictérina	PM	Biguglia, Gradugine	2	-	-	-	-	-	-	-	-	-	-	-	-	-	-	-	-
Hirondo rustica	PM MB	Biguglia, Gradugine	177	-	-	-	-	-	-	-	-	-	-	-	-	-	-	-	37/1[T‡]
Hirundo rustica X Delichon urbicum	hybrid	Biguglia	1	-	-	-	-	-	-	-	-	-	-	-	-	-	-	-	-
Luscinia megarhynchos	MB PM	Biguglia, Gradugine	15	-	-	-	-	-	-	-	-	-	-	-	-	-	-	1/1[R‡]	-
Luscinia svecica	PM WV	Biguglia	2	-	-	-	-	-	-	-	-	-	-	-	-	-	-	-	-
Motacilla flava	PM OB	Biguglia, Gradugine	4	-	-	-	-	-	-	-	-	-	-	-	-	-	-	-	-

Phoenicurus phoenicurus	PM	Biguglia, Gradugine	2	-	-	-	-	-	-	-	-	-	-	-	-	-	-	-	-
Phylloscopus collybita	PM WV OB	Biguglia, Gradugine	0	-	-	-	-	-	-	-	-	-	-	-	-	-	-	-	-
Phylloscopus sibilatrix	PM	Biguglia	0	-	-	-	-	-	-	-	-	-	-	-	-	-	-	-	-
Phylloscopus trochilus	PM	Biguglia, Gradugine	1	-	-	-	-	-	-	-	-	-	-	-	-	-	-	-	-
Prunella modularis	WV PM	Biguglia, Gradugine	1	-	-	-	-	-	-	-	-	-	-	1/0	-	-	1/0	-	-
Remiz pendulinus	PM WV	Gradugine	0	-	-	-	-	-	-	-	-	-	-	-	-	-	-	-	-
Riparia riparia	PM	Gradugine	5	-	-	-	-	-	-	-	-	-	-	-	-	-	-	-	-
Saxicola rubetra	PM OB	Biguglia	1	-	-	-	-	-	-	-	-	-	-	-	-	-	-	-	-
Sylvia borin	PM	Biguglia, Gradugine	27	-	-	-	-	-	-	-	-	-	-	-	-	-	-	-	-
Sedentary/Migratory																			
Alcedo atthis	PM WV RB	Gradugine	1	-	-	-	-	-	-	-	-	-	-	-	-	-	-	-	-
Buteo buteo	RB PM	Gradugine	2	-	-	-	-	-	-	-	-	-	-	-	-	-	-	-	-
Carduelis carduelis	RB PM	Gradugine	1	-	-	-	-	-	-	-	-	-	-	-	-	-	-	-	-
Cettia cetti	RB PM WV	Biguglia, Gradugine	95	-	-	-	1/1[Rh ‡]	-	-	-	-	-	-	-	-	-	3/0	-	-
Circus aeruginosus	PM RB WV	Gradugine	1	-	-	-	-	-	-	-	-	-	-	-	-	-	-	-	-

Cisticola juncidis	RB PM	Gradugine	0	-	-	-	-	-	-	-	-	-	-	-	-	-	-	-	-
Erithacus rubecula	RB PM WV	Biguglia, Gradugine	68/1^{Ec}	-	1/0	1/0	-	-	-	-	-	1/0	-	-	1/1$^{Rh\ \ddagger}$	1/0	4/1$^{Rh\ \ddagger}$	3/1$^{Ec\ \ddagger}$	-
Falco tinnunculus	RB PM WV	Gradugine	1	-	-	-	-	-	-	-	-	-	-	-	-	-	-	-	-
Fringilla coelebs	RB PM WV	Biguglia, Gradugine	7	-	-	-	-	-	-	-	-	-	-	-	-	-	-	-	-
Jynx torquilla	RB PM	Biguglia	4	-	-	-	-	-	-	-	-	-	-	-	-	-	-	-	-
Otus scops	RB PM	Biguglia, Gradugine	0	-	-	-	-	-	-	-	-	-	-	-	-	-	-	-	-
Passer italiae	RB PM	Gradugine	1	-	-	-	-	-	-	-	-	-	-	-	-	-	-	-	-
Saxicola rubicola	PM WV RB	Gradugine	1	-	-	-	-	-	-	-	1/1$^{R\ \ddagger}$	-	-	-	-	-	-	-	-
Sylvia atricapilla	RB PM WV	Biguglia, Gradugine	148	-	-	-	-	-	-	-	-	-	-	-	-	-	1/1$^{Ap\ \ddagger}$	-	-
Turdus merula	RB PM WV	Biguglia, Gradugine	17	-	-	1/0	-	-	-	-	-	-	-	-	-	-	2/1$^{R\ \ddagger}$	-	-
**Total**			**762/2^{WNV‡}, 1$^{Ec\ \ddagger}$**	**-**	**1/0**	**2/0**	**1/1$^{Rh\ \ddagger}$**	**-**	**-**	**-**	**2/1$^{R\ \ddagger}$**	**2/0**	**-**	**1/0**	**1/0**	**1/0**	**11/1$^{Ap\ \ddagger}$, 1^{R‡}, 1^{Rh‡}**	**4/ 1^{Ec‡}, 1^{R‡}**	**37/1^{T‡}**
